# Implementation of an interdisciplinary research model at a tertiary University Hospital in São Paulo, Brazil: post-COVID-19 cohort study

**DOI:** 10.1016/j.clinsp.2026.100925

**Published:** 2026-04-15

**Authors:** Laura Sampaio de Moura Azevedo, Marina Pires do Rio Caldeira, Moises Victor Ribeiro Nunes, Carolina Monteiro Lobato Del Villar, Denise Lungwtz Ramalho, Thais Suemi Yokoyama, Pedro Rizzi de Oliveira, Thais Mauad, Orestes Vicente Forlenza, Linamara Rizzo Battistella, Emmanuel A. Burdmann, Marcia Thereza Couto, Carlos Roberto Ribeiro Carvalho

**Affiliations:** a Faculdade de Medicina da Universidade de São Paulo, São Paulo, SP, Brazil; b Divisão de Pneumologia, Instituto do Coração (InCor), Hospital das Clínicas da Faculdade de Medicina da Universidade de São Paulo (HCFMUSP), São Paulo, SP, Brazil; c Laboratorio de Neurociencias (LIM-27), Departamento e Instituto de Psiquiatria, Hospital das Clínicas da Faculdade de Medicina da Universidade de São Paulo (HCFMUSP), São Paulo, SP, Brazil; d Institute of Physical Medicine and Rehabilitation of General Hospital of Faculdade de Medicina da Universidade de São Paulo (FMUSP), São Paulo, SP, Brazil; e Departamento de Clínica Médica, LIM 12, Laboratório de Pesquisa Básica em Doenças Renais, Hospital das Clínicas da Faculdade de Medicina da Universidade de São Paulo (HCFMUSP), São Paulo, SP, Brazil; f Departamento de Medicina Preventiva, Faculdade de Medicina da Universidade de São Paulo (FMUSP), São Paulo, SP, Brazil

**Keywords:** Interdisciplinary research, Post-acute COVID-19 syndrome, Health management, Instruments for management of scientific activity, Clinical research protocol

## Abstract

This study describes the implementation of an interdisciplinary model within the COVID-19 cohort at Hospital das Clínicas, Universidade de São Paulo (HCFMUSP). Among 1957 survivors, 749 attended the first follow-up (6‒11 months), revealing that 83% reported persistent symptoms ‒ mainly fatigue, dizziness, pain, and dyspnea ‒ highlighting the need for extended monitoring. A four-year program involving 22 study groups was established to characterize symptom frequency, severity and disability, explore clinical and sociodemographic associations, and evaluate the potential for recovery through multidisciplinary approaches. A dedicated team coordinated recruitment, teleconsultations, in-person assessments and database management. Of 749 patients, 523 completed teleconsultations, 433 attended the first visit, and 367 the second. The implementation demonstrated that interdisciplinary research enhances patient engagement, reduces fragmentation, and supports efficient and high-quality research in complex human-based studies.

## Highlights

• Interdisciplinary model assessed over 350 post-COVID patients.
• Over 30 assessments generated ∼11,000 patient evaluations.
• Integrated dataset strongly supports interdisciplinary research.
• Hybrid model optimized patient time and study logistics.

## Introduction

COVID-19, first identified in December 2019, quickly escalated into a pandemic with significant global health consequences. In January 2020, the World Health Organization (WHO) declared it a Public Health Emergency of International Concern, and by March, it was classified as a pandemic.^1^^,^^2^ Throughout the pandemic, many individuals infected with SARS-CoV-2 developed persistent, often disabling symptoms, impairing quality of life and affecting multiple organ systems as well as physical, cognitive, and mental health.^3^, ^4^, ^5^, ^6^, ^7^, ^8^, ^9^, ^10^ The monitoring and treatment of these long-term symptoms and sequelae emerged as a major global public health challenge.^3^, ^4^, ^5^^,^^8^, ^9^, ^10^, ^11^

In this context, a cohort named the COVID-19 Study Group of hospitalized COVID-19 survivors was established to characterize their clinical features and persistent post-discharge symptoms, and to identify associated clinical, sociodemographic, and environmental factors. The initiative was made possible through a multidisciplinary, collaborative project supported by civil society donations.^12^ Post-COVID-19 sequelae assessments were conducted 6–11-months after hospital discharge^12^^,^^13^ and again 18–24-months after discharge,^9^ the latter involving only a subsample of the patients.

Conducted six to eleven months after hospital discharge, the initial follow-up comprised physical examinations, mental health evaluations, and assessments of disability, quality of life, and physical functioning. Among nearly 3000 patients, 1957 survived hospitalization, and 749 participated in this first follow-up (2020–2021).^12^^,^^13^ Given that this assessment took place in 2020–2021 ‒ when limited knowledge was available about the disease’s acute and chronic phases ‒ the evaluation was organized in a multiprofessional format, with each researcher focusing on outcomes specific to their area of expertise.^12^

These initial evaluations revealed that 618 patients (83%) continued to experience at least one symptom 6–11 months after discharge, most commonly fatigue, dizziness, body pain, and dyspnea.^10^ Twenty percent of the patients reported having 1 or 2 persistent symptoms, 26% had 3‒5 symptoms, and 45% of participants had more than 5 symptoms. Only 9% of the study group reported having no post-COVID-19 symptoms.^11^

Since COVID-19 affects multiple organs, it is crucial to develop scalable and interdisciplinary healthcare models for better understanding and managing both acute and post-COVID-19 phases.^8^, ^9^, ^10^^,^^12^, ^13^, ^14^ The pandemic also exposed structural limitations in clinical research, especially the fragmentation caused by subspecialization, emphasizing the need to break down disciplinary silos.^15^ Overcoming disciplinary fragmentation is a structural challenge within academic and clinical research that requires institutional arrangements that promote interdisciplinary research.^16^

Interdisciplinary Research (IDR) is defined as “a mode of research by teams or individuals that combines information, data, techniques, tools, perspectives, concepts, and/or theories from two or more disciplines or bodies of specialized knowledge to enhance fundamental understanding or to solve problems whose solutions are beyond the scope of a single discipline or area of research practice”.^16^

This approach promotes patient-centered research, facilitates participation, boosts engagement, encourages knowledge sharing, and broadens access to expertise, resources, and funding. By combining different perspectives to tackle complex challenges, IDR enhances scientific productivity, visibility, prestige, and research quality.^15^, ^16^, ^17^, ^18^

Therefore, to improve efficiency and generate robust evidence, clinical research ‒ like clinical care ‒ must incorporate expertise across specialties, ensuring that relevant information is widely available and accessible. Drawing on the collective knowledge of different disciplines enables collaborative models that foster integrated, high-quality care and continuous innovation in healthcare delivery.^15^

Within this framework, the COVID-19 Study Group submitted a proposal to the São Paulo Research Foundation (FAPESP) to fund patient assessments over a four-year follow-up period, aiming to transition the cohort’s evaluation model from a multidisciplinary to an interdisciplinary approach. The proposal included 15 institutional disciplines targeting different patient groups at various time points during the 48-month period. Its goals were to describe the frequency and severity of symptoms and disability indices, investigate associations between persistent COVID-19 symptoms and clinical or sociodemographic variables, and assess the potential for reversing post- COVID sequelae through a multidisciplinary and interdisciplinary approach.

Thus, the objective of the present study was to describe a protocol of an interdisciplinary model for managing follow-up assessments of medium- and long-term sequelae in patients with moderate to severe COVID-19 who survived hospitalization at Hospital das Clínicas da Faculdade de Medicina da Universidade de São Paulo (HCFMUSP). Additionally, the authors aimed to assess how coordination across project stages, including teleconsultations, in-person visits, and medical feedback sessions, can influence patient adherence.

## Materials and methods

### Study design

This study was the third follow-up of the COVID-19 Study Group cohort study of COVID-19 patients at HCFMUSP, which assessed patients 41‒47 months after discharge. It included adult patients with a positive RT-PCR test for SARS-CoV-2 between March 30 and August 31, 2020, approved by the Research Ethics Committee of the institution (n° 73166123.1.0000.0068). This study was reported in accordance with the Strengthening the Reporting of Observational Studies in Epidemiology (STROBE) guidelines.

### Assessment schedules

**Fig. 1:**
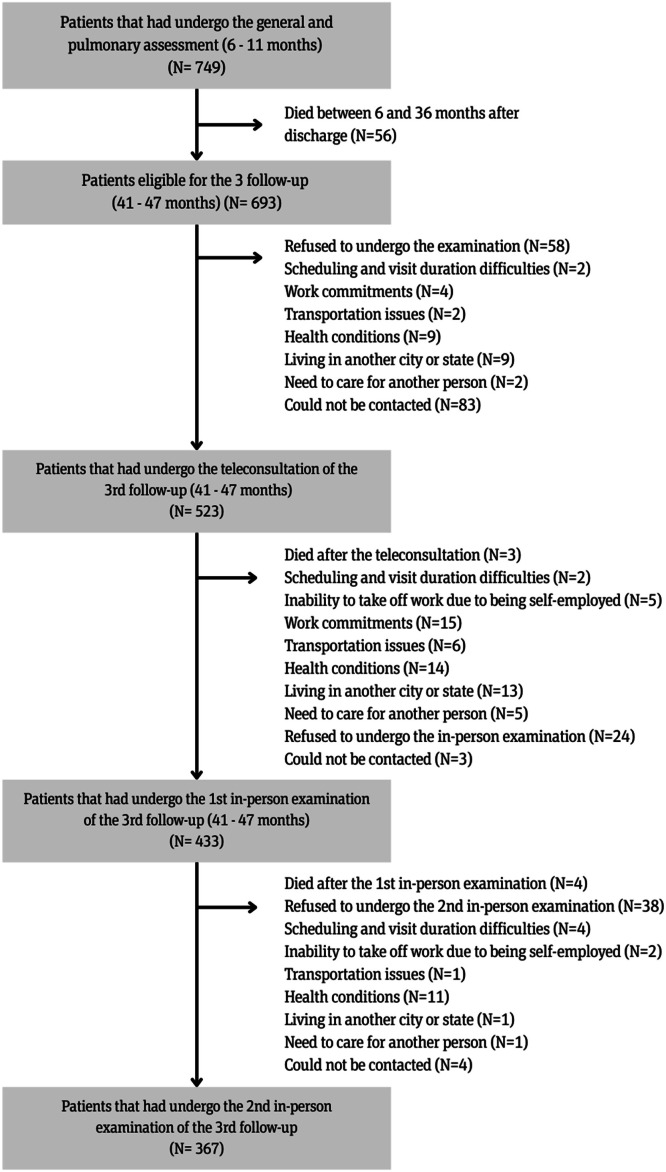
Flow-chart for selecting participating patients in the Follow-Up (FUP).

Among the 749 surviving patients who attended the first follow-up, 693 were eligible for the third follow-up at 41‒47 months after discharge, and 523 were included in the study and participated in the teleconsultation at 41‒47 months after discharge. Of these, 433 underwent the first in-person examination and 367 the second in-person examination (Fig. 1). Furthermore, the protocol included the synthesis of clinical information and the delivery of feedback sessions to the 330 patients who participated in the teleconsultations and subsequent in-person visits.

The main reasons reported by patients for not participating on the study were mostly related to scheduling and visit duration difficulties (*n* = 15), inability to take time off work due to being self-employed (*n* = 5), work commitments (*n* = 21), transportation issues (*n* = 13), health conditions (*n* = 33), living in another city or state (*n* = 20), the need to care for another person (*n* = 8), or lack of interest without a reported justification (*n* = 99).

### COVID-19 study group

An interdisciplinary collaboration network ‒ referred to as the COVID-19 Study Group ‒ was established, bringing together 22 study groups across 15 disciplines (Fig. S1)

**Fig. 2:**
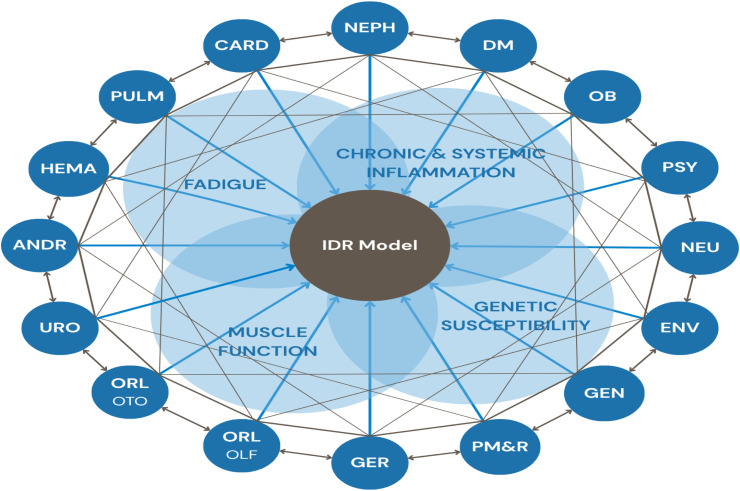
COVID-19 Study Group diagram illustrating the IDR model's structure. The light black connecting lines delineate the interrelations and interdependencies among disciplines, underscoring the multisystemic nature of post-COVID-19 conditions. The blue lines indicate each discipline’s contribution to the planning and implementation of the IDR model, positioned at the center of the diagram. The light blue circles represent cross-disciplinary themes ‒ chronic and systemic inflammation, genetic susceptibility, muscle function, and fatigue ‒ that span multiple disciplines and exemplify the integrative and collaborative interactions characteristic of the interdisciplinary approach. IDR, Interdisciplinary research; PULM, Pulmonology; CARD, Cardiology; NEPH, Nephrology; DM, Diabetes; OB, Obesity; PSY, Psychiatry; NEU, Neurology; ENV, Environment; GEN, Genetics; PM&R, Physical Medicine and Rehabilitation; GER, Geriatrics; ORL, Otorhinolaryngology; OLF, Olfaction; OTO, Otology; URO, Urology; ANDR, Andrology; HEMA, Hematology.

This model introduces a governance structure and a rigorous research protocol designed to guide the systematic investigation and understanding of the long-term consequences of SARS-CoV-2 infection, fostering connections among multiple clinical and research specialties in pursuit of a holistic investigative approach (Fig. 2).

The project involved more than 100 participants, including 73 researchers, 30 undergraduate and graduate students, 6 administrative staff, 5 professionals of the executive team and Faculdade de Medicina da Universidade de São Paulo (FMUSP)’s institutional project support office, responsible for the project structure, administrative implementation, and financial management.

### Establishment of an executive team

Due to the project's complexity ‒ which involved recruiting approximately 700 patients, managing both remote and in-person appointments, conducting multiple tests and sample collections, and coordinating the work of more than 100 participants ‒ a dedicated implementation team was established.

This team consisted of five professionals, including one project coordinator, three study coordinators, and one data manager, led by the principal investigator. The team was responsible for the planning, structuring, and execution of the follow-up.

The key activities of the implementation team included recruiting and scheduling patients, conducting teleconsultations, coordinating the workflow and execution of in-person assessments, managing and updating the database, supporting researchers in resolving pending issues, and adapting the protocol as needed.

During the planning process, the dedicated implementation team was trained on administering assessment scales during teleconsultations, procedures for obtaining the Informed Consent Form (ICF), and conducting specific in-person assessments.

Besides the executive team, there was Faculdade de Medicina da Universidade de São Paulo (FMUSP)’s institutional project support office that was responsible for purchasing, paying services, submitting research scholarship requests to FAPESP, and preparing scientific and financial reports, given that its funding exceeded U$1900,000.00.

### Coordination meetings

Coordination meetings were held with all involved researchers. Key discussion topics included:

1. Presentation of care flow proposals.
2. Confirmation of assessments and exams to be conducted.
3. Proposals for optimizing laboratory tests and aliquots to be collected.
4. Operational status updates.
5. Identification of barriers and bottlenecks in workflow, followed by the joint development of action plans.
6. Presentation of initiatives to optimize visits and improve the patient experience.
7. Structuring and dissemination of the data request protocol.
8. Resolution of pending issues.
9. Dissemination of manuscripts published by the group.
10. Guidance on proper citation and acknowledgments.

These meetings were scheduled to organize the assessment workflows for in-person visits, define the set of evaluations and laboratory tests to be collected and ensure data entry into the CRF. Through the alignment of planned examinations, it became possible to identify tests of shared interest across disciplines and, consequently, harmonize protocols so that a single assessment could meet the needs of multiple research groups. For example, while the Physical Medicine and Rehabilitation team initially planned to perform the 10-meter walk test and the Pulmonology group intended to conduct the 6-minute walk test, the alignment meetings led to the agreement to adopt only the 6-minute walk test, thereby accommodating both disciplines’ objectives.

This process aimed to optimize the volume of blood drawn and evaluation procedures, reducing patients’ time spent in the hospital. In this sense, the interdisciplinary model minimized patient discomfort by avoiding redundant procedures, ultimately enhancing the patient care experience, improving operational efficiency, and facilitating participant engagement in the study.

During these meetings, the development of manuscripts was also discussed, including the sharing of planned manuscripts by the subprojects as well as proposals for collaborative manuscripts. The aim was to promote a comprehensive view of the patient and encourage the sharing and integration of knowledge.

### Integrated assessment pathway

**Fig. 3:**
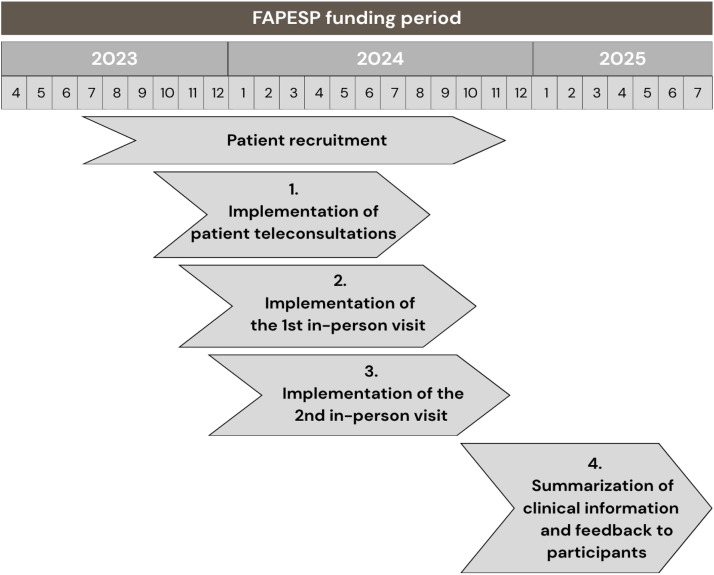
Timeline of the HCFMUSP COVID Cohort Study. FAPESP, The São Paulo Research Foundation.

The assessment was organized into four phases: 1) Teleconsultation, 2) First in-person assessment, 3) Second in-person assessment, and 4) Summarization of clinical information and feedback to participants. The implementation followed a gradual timeline that started in October 2023 and ended in July 2025 (Fig. 3).

During the four months before the assessments began, patients were contacted to inform them about the start of the follow-up and to confirm their willingness to continue participating in the study. Among the 749 patients who took part in the first follow-up, 693 survived and were eligible to participate in the follow-up.

Phase 1 ‒ Teleconsultation

Appointments were scheduled through messages sent via the project's WhatsApp, calls made from the institutional phone, and during the conclusion of the previous follow-up stage. After scheduling, patients received a message with confirmation and instructions for the assessments. For in-person visits, the core team confirmed appointments 48 h in advance and, in cases of cancellation, sought to reschedule another patient.

The teleconsultation was carried out through 50-minute video calls using iConf, a platform developed and validated at the Instituto do Coração of HCFMUSP.^20^^,^^21^ It involved the use of an identification form that collected sociodemographic characteristics, employment history, retirement status (pre- and post- COVID-19), self-reported medical and health history, focusing on past and current morbidities, medication use, COVID-19 vaccination, and hospitalization history. Additionally, 20 standardized scales were used to assess dyspnea, functional status, disability, frailty, fatigue, functional independence, immunological symptoms, sleepiness and insomnia, health-related quality of life, diet, vision, hearing and smell, urinary incontinence and other urological and nephrological symptoms, female sexuality, neuropathy, cardiovascular risk, food insecurity, screening for neuromuscular diseases, and questions regarding allergic reactions to contrast agents used in pulmonary artery CT angiography screening.

Phase 2 – 1st In-person visit

The 1st in-person visit occurred at the Prof. Dr. Fúlvio Pileggi Research Center situated at InCor HCFMUSP. Patients arrived at 12:00 pm. and were discharged at 6:00 pm. During the initial visit, the Informed Consent Forms were signed, vital signs, anthropometric data, blood and urine samples were collected, and an electrocardiogram was performed. Serum and plasma aliquots were stored at the institutional biobank for later analysis of antibodies, miRNAs, cytokines, interleukins, and other plasma markers. Additional assessments included pulmonary function tests, ultrasound of the *rectus femoris, vastus intermedius*, and diaphragm muscles, the DN4 questionnaire for diagnosing neuropathic pain, the Brief Pain Inventory, respiratory muscle strength tests (PIMax, PEMax, SNIP), the 1-minute sit-to-stand test, Timed Up & Go, handgrip strength test, six-minute walk test, and the objective olfactory test using the NOAR® MultiScent device.

**Fig. 4:**
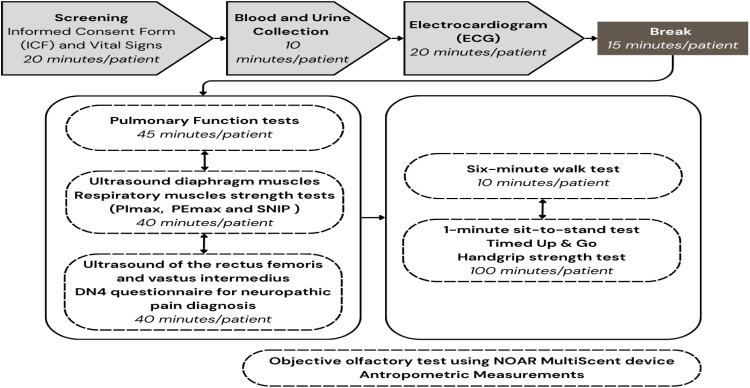
Reviewed the initial in-person evaluation flow in the Follow-Up (FUP). PImax, Maximal Inspiratory Pressure; PEmax, Maximal Expiratory Pressure; SNIP, Sniff Nasal Inspiratory Pressure; DN4, Douleur Neuropathique-4.

It was established that the assessments would follow a sequential circuit, where each patient would start with one examination and, as each assessment was completed, would be guided to the subsequent evaluations, following only the premise that the ultrasounds and pulmonary function assessments needed to be conducted before the functional tests and the 6-minute walk test (Fig. 4). The objective olfactory test using the NOAR® MultiScent device and anthropometric measurements was performed depending on the patient’s availability.

Phase 3 – 2nd In-person visit

**Fig. 5:**
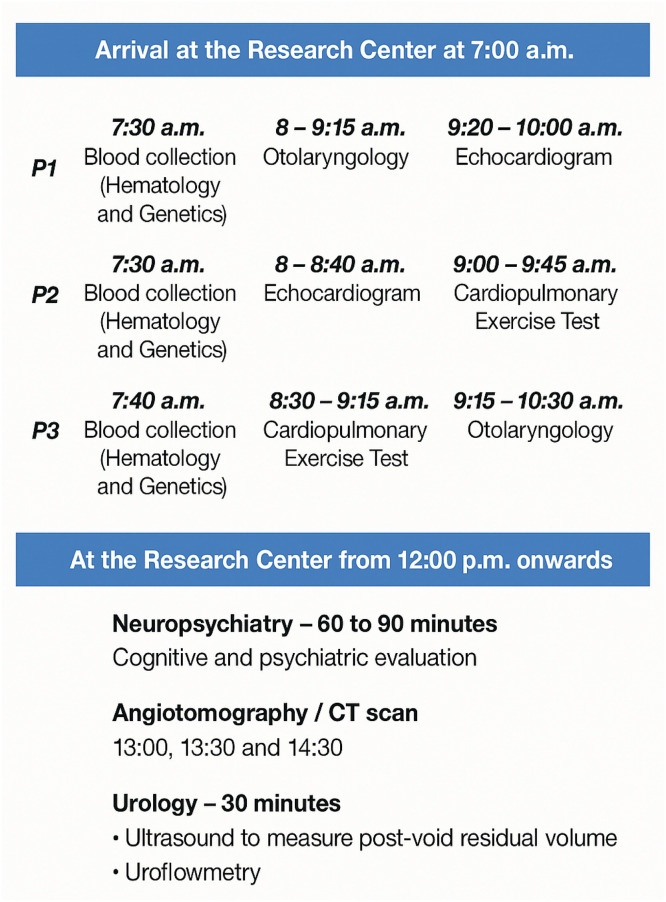
Reviewed 2nd in-person evaluation flow in the Follow-Up (FUP). P1 (1st Patient), P2 (2nd Patient), P3 (3rd Patient).

During the second in-person visit, patients arrived at 7:00 a.m. and were discharged at 3:00 pm. They underwent evaluations with the otorhinolaryngology and audiology teams, along with hematological and genetic tests, an echocardiogram, cardiopulmonary exercise testing, a chest CT scan, pulmonary artery CT angiography. They also received psychiatric and cognitive assessments, an ultrasound for post-void residual volume measurement, and uroflowmetry. Pulmonary artery CT angiography was conducted using the Aquilion One Prism™ CT scanner – Canon Medical Systems Corporation, Tokyo, Japan (Fig. 5).

After completing the second in-person visit, patients who were available and interested were invited to undergo 24-hour Holter monitoring, which was installed after the evaluations and removed the next day. Additionally, after visits 1 and 2, the neuropsychiatry, andrology, and cardiology teams contacted patients to schedule specific exams, such as brain PET/MRI, scrotal ultrasound with Doppler, semen analysis, administration of the 15-question International Index of Erectile Function (IIEF) Questionnaire scale and other questionnaires, cardiac Magnetic Resonance Imaging, and the Tilt Test.

Phase 4 – Summarization of clinical information and feedback

After completing the in-person visits, scheduled sessions were conducted to summarize clinical information and provide feedback to participants through teleconsultation. The primary objectives were to give patients detailed explanations of their test results and discuss any identified sequelae, if present. Additionally, the sessions aimed to plan ongoing follow-up care to monitor patients' health progression as needed. These sessions were held via the IConf platform, offering a secure and accessible environment for remote care. Each session lasted approximately 20 minutes. Following the feedback sessions, when relevant changes were identified, additional consultations and/or examinations were scheduled.

### Patient engagement

To improve patient engagement, the study team implemented several strategies. First, participants received a confirmation message 24 hours before their scheduled appointments, which reinforced adherence and reduced absenteeism. In addition, teleconsultations for the administering assessment scales were adopted to decrease both the time spent at the hospital and the number of required in-person visits, thereby improving adherence. Another strategy involved summarizing clinical information and holding dedicated feedback sessions, during which the physician presented examination results and addressed patients’ questions. This approach fostered a sense of support and care, increasing participants’ willingness to remain in the study.

### Data collection, management, and quality control

Study data include sociodemographic information, race, educational level, hospitalization characteristics, clinical data, return to employment status, and COVID-19 vaccination. All clinical data were organized in a structured form using REDCap software (https://www.redcapbrasil.com.br/).^21^ A copy of the Case Report Form is provided as online supplemental material. A REDCap team will manage the database and grant research groups access for interim and final analyses.

## Results

During the in-person visits, approximately 11,000 examinations were performed by the research team. Assessments conducted during the follow-up are shown in Table 1.

**Table 1:** Assessments conducted during the in-person assessments.

Examinations	n (%)
Vital signs and anthropometric data	432 (99.8)
Measurement of vital signs using the digital device *MTX Cnoga®*	427 (98.6)
Blood tests	432 (99.8)
Urine tests	420 (97.0)
Objective olfactory test using the *NOAR MultiScent* 20 device	392 (90.5)
Electrocardiogram (ECG)	424 (97.9)
Plethysmography and oscillometry	393 (90.8)
Ultrasound (USG) of the diaphragm and parasternal intercostal muscle, and assessment of ventilatory muscle strength	306 (70.7)
Sit-to-stand test (WHO protocol)	431 (99.5)
1-minute sit-to-stand test	431 (99.5)
Six-minute walk test with holter oximetry	363 (83.8)
Visual analog scale (VAS) for pain	414 (95.6)
Muscle strength – MRC Scale	427 (98.6)
Timed up and go (TUG) test	420 (97.0)
Handgrip strength test	428 (98,8)
DN4 questionnaire for neuropathic pain screening	291 (67.2)
Brief pain inventory (BPI)	421 (97.2)
Ultrasound of the rectus femoris and vastus intermedius muscles	430 (99.3)
Genetic assessment	330 (76.2)
Hematologic assessment	330 (76.2)
Cardiopulmonary exercise test (CPET)	219 (50.6)
Chest computed tomography (CT)	350 (80.8)
Pulmonary artery CT angiography	216 (49.9)
Cognitive assessment	360 (83.1)
Psychiatric assessment	355 (82.0)
Uroflowmetry	330 (76.2)
Ultrasound for post-void residual measurement	351 (81.1)
Echocardiogram	362 (83.6)
24-hour holter monitoring	115 (26.6)
Otology assessment	247 (57.0)
Total	10.847

The interdisciplinary model proved essential for ensuring the breadth and depth of data collection throughout the cohort. This approach enabled the acquisition of information on vital signs, anthropometric parameters, and blood and urine tests from more than 420 patients (97%), as well as cardiological assessments ‒ including electrocardiogram and echocardiogram results ‒ from over 360 patients (83%).

Pulmonary evaluations were also carried out extensively: 219 participants (50%) completed dynamic tests such as the Cardiopulmonary Exercise Test (CPET), sit-to-stand test (WHO protocol), one-minute sit-to-stand test, and six-minute walk test with Holter oximetry, while 393 (90%) underwent plethysmography and oscillometry.

Moreover, 211 patients (49%) completed ultrasound assessments of the rectus femoris and vastus intermedius muscles, as well as the diaphragm and parasternal intercostal muscles, in addition to ventilatory muscle strength testing, chest computed tomography, and handgrip strength measurement.

Some assessments could not be performed on patients due to health conditions that either prevented or contraindicated their execution. Additionally, because of the delay in acquiring the ultrasound equipment, the ultrasound of the diaphragm and parasternal intercostal muscles and ventilatory muscle strength assessments began only in January 2024. Consequently, around 125 patients did not undergo these evaluations, as shown in Table 1.

Furthermore, 24-hour Holter monitoring required patients to return the next day, which limited the number of participants able to complete the test to less than half. Over the course of the project, the otology assessment was conducted only twice a week, resulting in approximately 30% of patients not undergoing the evaluation, as shown in Table 1.

Equipment maintenance requirements or technical difficulties prevented the measurement of blood pressure, heart rate, oxygen saturation, cardiac output, stroke volume, cardiac index, PCO_2_, and PO_2_ using the MTX Cnoga® digital device, as well as the objective olfactory test with the NOAR MultiScent 20® device, in 6 and 41 patients, respectively, as shown in Table 1.

## Discussion

Considering that a substantial proportion of survivors of COVID-19 disease have persistent and multisystem symptoms, understanding and managing these long-term sequelae poses a major public health challenge. In response, the COVID-19 Study Group established a large cohort of previously hospitalized patients to characterize post-discharge symptoms and identify associated clinical, sociodemographic, and environmental factors.

The IDR model adopted in the COVID-19 Study Group cohort was particularly well-suited to this context. By integrating 22 study groups within a unified structure, the model represents a critical effort to advance understanding of post-COVID-19 trajectories and to support the development of scalable, interdisciplinary models for long-term care and research. As such, the study supported the formulation of broad and clinically relevant research questions that could not be adequately addressed by individual disciplines.

The coordinated completion of approximately 11,000 examinations required continuous communication among research teams and investigators and careful planning and coordination to optimize patients’ time spent in the hospital for the study. Since all assessments were conducted over only two days, scheduling negotiations regarding the timing and duration of each evaluation were essential to prevent overlap or interference between procedures.

For instance, on the first day, it was necessary to separate assessments that required physical exertion from those that could be affected by prior physical activity. On the second day, the initially proposed schedule required revision, as waiting times for the CT scan were delaying other morning assessments. This occurred because the CT examinations for the study were performed in the same setting used for inpatient and outpatient imaging, resulting in competition with the hospital’s routine clinical demand. Therefore, the team decided to reschedule the echocardiography to the morning period and move the CT scan to the afternoon.

It also enabled the integration of findings into a single database accessible to all researchers in the group, thus facilitating the development of studies that describe the involvement of individual organs and systems. Most importantly, it supports interdisciplinary studies that promote understanding of SARS-CoV-2 dissemination and its overall impact on the body.

One of the most evident benefits of the IDR model was its ability to harmonize protocols and reduce redundancies in assessments. Regular coordination meetings and ongoing communication among researchers allowed the identification of tests desired by multiple disciplines and their consolidation into a single procedure ‒ for example, the walk test initially proposed in different formats by distinct research groups. This integration reduced the volume of blood drawn, avoided duplicate tests, and shortened the time participants spent in the hospital. These outcomes are consistent with the literature showing that interdisciplinary approaches increase efficiency, prevent waste, and optimize human resources, particularly in large-scale projects.^15^

Also, the IDR model enabled cross-disciplinary collaboration, supporting the development of studies that examined systemic interactions and multisystem outcomes. From the interaction among the multiple study groups, several cross-disciplinary lines of inquiry emerged, with the most salient encompassing fatigue, chronic and systemic inflammation, muscle function, and genetic susceptibility. Fatigue is being characterized through blood biomarkers, plethysmography and oscillometry, the six-minute walk test with Holter oximetry, standardized scales, and cytokine and interleukin profiling. Chronic and systemic inflammation is being assessed through cytokine and interleukin profiling and is being examined in relation to the development of diabetes and obesity, as well as to sequelae affecting multiple organ systems. Muscle function is being described through blood tests, plethysmography, Ultrasound (USG) of the diaphragm, parasternal intercostal, rectus femoris, and vastus intermedius muscles, ventilatory muscle strength assessment, Timed Up and Go (TUG) test, and handgrip strength measurement. Genetic analyses were also performed to identify variants associated with susceptibility to pulmonary sequelae and diabetes. Additional analyses investigated associations between urinary symptoms and metabolic syndrome; the prevalence of sexual dysfunction in women with long COVID and its association with anosmia; and the influence of environmental factors on cognitive, psychiatric, renal, and pulmonary sequelae.

When comparing the assessment protocol of the first follow-up (6–11 months after discharge) with the current follow-up, important continuities and structural improvements become evident. Both follow-ups required three patient contacts with the hospital ‒ an initial remote stage followed by in-person assessments and a summarization of clinical information and feedback. However, the scope and methodological rigor of the procedures diverged substantially.

In the first follow-up, the remote stage focused on lifestyle habits, physical activity, self-rated health, and an extensive symptom review across multiple specialties. The in-person stage included laboratory tests, simple spirometry, the sit-to-stand test, olfactory assessments, muscle ultrasound, neurological, cognitive, and psychiatric evaluations, medical interviews, and chest radiography. A subset of patients ‒ those requiring intensive care during hospitalization or presenting symptoms suggestive of pulmonary dysfunction ‒ were invited for an additional visit to undergo chest CT, plethysmography, and cardiopulmonary exercise testing. As a result, completing the full protocol required two hospital visits, similar to the current follow-up.

However, the present follow-up demonstrates a marked enhancement in scope and integration, largely attributable to the Interdisciplinary Research (IDR) model adopted for the cohort. By aligning protocols across 22 study groups, the team was able to consolidate procedures, reduce redundancies, and substantially expand the number and diversity of assessments conducted within the same two-visit structure.

This comparison illustrates how the IDR model allowed the research team to conduct a broader, more comprehensive, and methodologically integrated set of evaluations without increasing the burden on participants. By fostering coordination across disciplines and streamlining assessment workflows, the model enhanced operational efficiency and strengthened the study's capacity to capture the multisystemic nature of post-COVID-19 sequelae ‒ an outcome that would have been unlikely under a traditional disciplinary approach.

By gathering leading researchers at FMUSP and HCFMUSP, securing support from a state Research Foundation and a donation from an individual managed by Fundação Faculdade de Medicina, and utilizing the project’s financial office, it was possible to implement an interdisciplinary model for managing the cohort study conducted at HCFMUSP on post-acute COVID-19 assessment. This interdisciplinary model, unique in the Institution, enabled the four-year follow-up of patients who survived severe COVID-19 hospitalization, focusing on patient-centered care and aiming to obtain the maximum amount of data during the two-day on-site evaluation.

Together, these evaluations reflect the study’s integrative design, which sought not only to document isolated physiological outcomes but to explore the complex interplay between organ systems affected by SARS-CoV-2 infection. By combining cardiopulmonary, muscular, and metabolic data within a single analytical framework, the interdisciplinary model allowed for a comprehensive characterization of post-COVID-19 sequelae ‒ highlighting the added value of collaborative, patient-centered research approaches in understanding long-term recovery trajectories.

In line with previous research, this study encountered challenges in reducing loss to follow-up.^22^, ^23^, ^24^, ^25^, ^26^, ^27^, ^28^ Of the 693 eligible patients, 523 participated, and 367 completed the 4-year follow-up, resulting in a 47% attrition rate.

Consistent with the Brazilian Birth Cohorts Consortium (Ribeirão Preto, Pelotas, and São Luís),^22^ the main challenges in maintaining follow-up included losing contact due to changes in address and phone numbers, as well as participants refusing to continue the assessments.

To reduce the loss to follow-up, continuous contact was maintained with participants. Additional contact details from family members and friends were collected, and teleconsultations, as well as the summarization of clinical information and feedback to participants, were implemented as strategies to enhance engagement and recognize their contribution to the study.

Across international prospective cohorts, follow-up of COVID-19 survivors has faced substantial attrition, although with considerable variation across settings. In Japan, Yagi et al.^23^ reported a 34% loss after 12-months, while in Thailand, 36.4% of eligible participants were excluded within one year for reasons including death, incarceration, foreign nationality, or direct loss to follow-up.^24^ The Korean adult cohort similarly experienced marked losses, with only 51.5% completing all four scheduled visits over two years.^25^ In contrast, the long-term Saudi Arabian cohort showed comparatively strong retention ‒ only 7.1% dropout over four years ‒ although participation declined steadily across time points.^26^ Severe retention challenges were documented in Israel, where a 75% nonresponse rate for the two-year questionnaire substantially increased the risk of bias.^27^ Likewise, the Wuhan cohort exhibited shrinking sample sizes across follow-ups, with only a fraction of participants completing pulmonary function tests, raising concerns about sampling bias.^28^

Therefore, even though the study included three assessment points ‒ one teleconsultation and two in-person visits ‒ and was conducted 41–47 months after discharge, the attrition rate is consistent with the literature.

Aside from loss to follow-up, one of the key challenges of the study was to develop a patient-centered sequential model that included all the assessments planned by the subprojects. Although the diversity of specialties and evaluations was the main strength of the study, it also increased the complexity of the evaluation flows during the in-person assessments.

The present findings may help improve understanding of the benefits of interdisciplinary collaboration in the conduct of human-based research. Furthermore, this study provides a foundation for developing a collaborative model that, through interdisciplinarity, enables interdisciplinary knowledge flow, access to specialized expertise, resources, and funding opportunities. Additionally, as described in the literature,^17^^,^^19^, ^20^, ^21^ this approach was associated with promoting patient-centered clinical research, as it facilitates participation and enhances patient engagement.

This initiative aims to foster cross-disciplinary integration within and across specialties and bring together diverse perspectives and resources. This approach addresses relevant research questions and promotes more effective, resource-efficient studies.

Van Rijnsoever and Hessels^17^ argue that interdisciplinary collaboration typically incurs higher coordination costs due to the cognitive distance between partners, which increases the demands for negotiation, alignment, and communication. They also note that funding schemes and evaluation systems are predominantly discipline-oriented, which may place interdisciplinary projects at a disadvantage when competing for resources compared to traditional, discipline-specific initiatives.

In the context of the present study, however, the implementation of the IDR model required only one additional resource: the executive team responsible for coordinating the project’s operational workflow. Considering the magnitude of the cohort, the substantial optimization of visits, and the expected scientific and clinical impact, this investment was deemed highly cost-effective.

Moreover, contrary to the disadvantages described by van Rijnsoever and Hessels,^17^ interdisciplinarity was a decisive factor in securing project funding. The proposal benefited from the integration of expertise across multiple specialties, bringing together highly experienced researchers from 22 groups and enabling a comprehensive, system-level approach to investigating post-COVID-19 sequelae. This collaborative structure strengthened the project’s scientific merit and contributed to its competitiveness in the funding process.

The study has several strengths. Most importantly, the interdisciplinary model facilitated collaboration among 15 disciplines and numerous complex evaluations, such as Computed Tomography Pulmonary Angiography (CTPA), chest Computed Tomography (CT), Cardiopulmonary Exercise Testing (CPET), plethysmography, and echocardiography, with a minor patient loss of around 10%. Over 300 patients attended both the teleconsultation and the two in-person visits to undergo more than 25 assessments across multiple specialties, providing a comprehensive and holistic view of each patient's post-COVID-19 condition.

Additionally, the interdisciplinary model enabled multiple evaluations to take place during a single 30-minute telehealth consultation and two in-person visits, each lasting 6 and 8 hours. This reduces hospital stays and prevents multiple hospital visits and repeated evaluations.

The implementation of the interdisciplinary model in the HCFMUSP cohort also generated important lessons. Reconciling schedules, aligning expectations across 22 groups, and adjusting operational workflows highlighted that although interdisciplinarity is highly beneficial, it requires careful structuring, clear governance mechanisms, and continuous communication. Adjustments made across phases ‒ such as reorganizing visit schedules, optimizing examination workflows, and refining strategies to reduce follow-up losses ‒ demonstrate that IDR models must be dynamic and adaptive.

Therefore, although interdisciplinarity stands out as one of the key strengths of this study, the requirement to undergo multiple assessments posed challenges to patient engagement, as participation demanded considerable time and commitment ‒ including a 50-minute teleconsultation and two in-person hospital visits.

This level of involvement may have contributed to participant fatigue and withdrawal. Still, given the study’s aim to explore the associations between persistent COVID-19 symptoms and clinical or sociodemographic variables, the adoption of an interdisciplinary model was essential. It enabled a more comprehensive understanding of the multifaceted nature of post-COVID-19 outcomes, which could not have been achieved through isolated disciplinary approaches.

Several operational lessons emerged from the 41–47-month follow-up that will guide the design of future assessments, including the upcoming 60-month evaluation. First, consolidating all procedures into a single visit was identified as a feasible strategy to improve patient adherence and satisfaction, as well as reducing patients’ transportation and meal costs. Although this approach requires patients to remain in the hospital for a longer period, many expressed a clear preference for a single extended visit rather than returning on a second day.

Also, during the follow-up, samples were obtained on two separate days, which generated discomfort and dissatisfaction among participants. Therefore, in future evaluation the logistics of blood collection will be optimized as it will adopt a single blood collection.

Finally, aligning the assessment schedule with the operational dynamics of diagnostic services proved essential. For instance, the decision to shift the CT scans to the afternoon unintentionally coincided with peak service hours, resulting in extended waiting times and participant complaints. Subsequent follow-ups will incorporate a more systematic analysis of institutional workflows to minimize bottlenecks and enhance the overall efficiency of patient assessments.

The limitations of the present study include the fact that 523 (75%) of 693 eligible patients participated in this follow-up, and 367 (70%) patients completed the whole protocol. However, when comparing sociodemographic and clinical characteristics of patients from the 6‒12 months after discharge follow-up who attended this follow-up with those who refused, the authors identified a slight bias in the cohort related to education level (Supplementary Table 1).

When comparing the sociodemographic and clinical characteristics collected at hospitalization and at the first follow-up (6–12 months after discharge) between post-COVID-19 patients who participated in the teleconsultation of the third follow-up (41–47 months) but did not complete all study stages and those who completed the teleconsultation as well as both in-person visits, the authors did not identify evidence of cohort bias (Supplementary Table 2).

Also, the study faced high absenteeism rates, approximately 35%, which extended the data collection period beyond the six months originally planned and ultimately contributed to loss to follow-up. To mitigate absenteeism, several engagement strategies were implemented, including reminder messages sent to participants, scheduling appointments according to patient availability, and offering assessment slots on nearly all weekdays ‒ except Wednesdays and weekends ‒ to maximize flexibility and participation.

## Conclusions

An interdisciplinary approach for managing follow-up assessments of medium- and long-term sequelae in patients with moderate or severe COVID-19 who survived hospitalization serves as a strategy to promote resource-conscious governance of financial and human resources, as well as patient-centered research.

Certainly, this integrated effort will contribute to generating novel data on the post COVID-19 condition, extending beyond medical specialties, improving clinical care, and generating a true collaborative atmosphere among clinicians and researchers in the Institution. Importantly, such an initiative improves the preparedness for the novel pandemics still to come.

Also, the implementation of the IDR model provides valuable recommendations for institutions seeking to implement similar approaches: invest in centralized coordination, promote ongoing dialogue among teams, and incorporate routines that balance scientific rigor, operational feasibility, and participant experience.

## Funding

This study was funded by the São Paulo Research Foundation (FAPESP) (grant number: 22/01769-5). Also, the project received a donation from an individual made through the Fundação Faculdade de Medicina.

## CRediT authorship contribution statement

**Laura Sampaio de Moura Azevedo:** Conceptualization, Methodology, Formal analysis, Data curation, Writing – original draft, Project administration. **Marina Pires do Rio Caldeira:** Conceptualization, Methodology, Resources, Project administration, Funding acquisition. **Moises Victor Ribeiro Nunes:** Investigation. **Carolina Monteiro Lobato Del Villar:** Investigation. **Denise Lungwtz Ramalho:** Resources, Project administration. **Thais Suemi Yokoyama:** Writing – review & editing. **Pedro Rizzi de Oliveira:** Writing – review & editing. **Thais Mauad:** Conceptualization, Methodology, Writing – review & editing, Project administration, Funding acquisition. **Orestes Vicente Forlenza:** Conceptualization, Methodology, Writing – review & editing, Project administration, Funding acquisition. **Linamara Rizzo Battistella:** Conceptualization, Methodology, Writing – review & editing, Project administration, Funding acquisition. **Emmanuel A. Burdmann:** Conceptualization, Methodology, Writing – review & editing, Project administration, Funding acquisition. **Marcia Thereza Couto:** Conceptualization, Methodology, Supervision. **Carlos Roberto Ribeiro Carvalho:** Conceptualization, Methodology, Writing – original draft, Writing – review & editing, Supervision, Project administration, Funding acquisition.

## Declaration of competing interest

The authors declare no conflicts of interest.

## Data Availability

The data that support the findings of this study are available on request from the corresponding author, CRRC.

## Appendix Supplementary materials

Image, application 1
