# Supplementary material for: Implementation of an interdisciplinary research model at a tertiary University Hospital in São Paulo, Brazil: post-COVID-19 cohort study
Source: Clinics (Sao Paulo). 2026 Apr 15;81:100925. doi: 10.1016/j.clinsp.2026.100925 (PMC13094481; doi:10.1016/j.clinsp.2026.100925)
Supplement: Supplementary file 1 [file mmc1.docx]

**CLINICS-D-25-01723**

**Supplemental Material**

# **Implementation of an interdisciplinary research model at a tertiary University Hospital in São Paulo, Brazil: Post-COVID-19 Cohort study**

Laura Sampaio de Moura Azevedo^1,2^, Marina Pires do Rio Caldeira^1^, Moises Victor Ribeiro^2^ Nunes, Carolina Monteiro Lobato Del Villar^2^, Denise Lungwtz Ramalho^1^, Thais Suemi Yokoyama^2^, Pedro Rizzi de Oliveira^2^, Thais Mauad^1^, Orestes Vicente Forlenza^3^, Linamara Rizzo Battistella^4^, Emmanuel A. Burdmann^5^, Carlos Roberto Ribeiro Carvalho [ORCID 0000-0002-1618-8509]^1,2^

^1^ Faculdade de Medicina da Universidade de Sao Paulo, Sao Paulo, SP, BR

^2^ Divisao de Pneumologia, Instituto do Coracao (InCor), Hospital das Clinicas HCFMUSP, Faculdade de Medicina, Universidade de Sao Paulo, Sao Paulo, SP, BR

^3^ Laboratorio de Neurociencias (LIM-27), Departamento e Instituto de Psiquiatria HCFMUSP, Faculdade de Medicina da Universidade de São Paulo

^4^ Institute of Physical Medicine and Rehabilitation of General Hospital of FMUSP Universidade de Sao Paulo, Sao Paulo, SP, BR

^5^ Departamento de Clinica Medica, LIM 12, Laboratorio de Pesquisa Basica em Doencas Renais, Hospital das Clinicas HCFMUSP, Universidade de Sao Paulo, Sao Paulo, Brazil

Corresponding author: carlos.carvalho@hc.fm.usp.br

| **Table of contents** | | **Page** |
| --- | --- | --- |
| **Supplementary figure 1** | COVID-19 Study Group diagram | 3 |
| **Supplementary table 1** | Comparison of hospitalization and first follow-up (6–12 months) sociodemographic and clinical variables between participants and non-participants of the third follow-up (41–47 months). | 4 |
| **Supplementary table 2** | Comparison of sociodemographic and clinical variables collected during hospitalization and at the first follow-up (6–12 months after discharge) between post-COVID-19 patients who participated in the teleconsultation of the third follow-up (41–47 months) but did not complete all study stages, and those who completed the teleconsultation as well as both in-person visits of the third follow-up. | 6 |

***
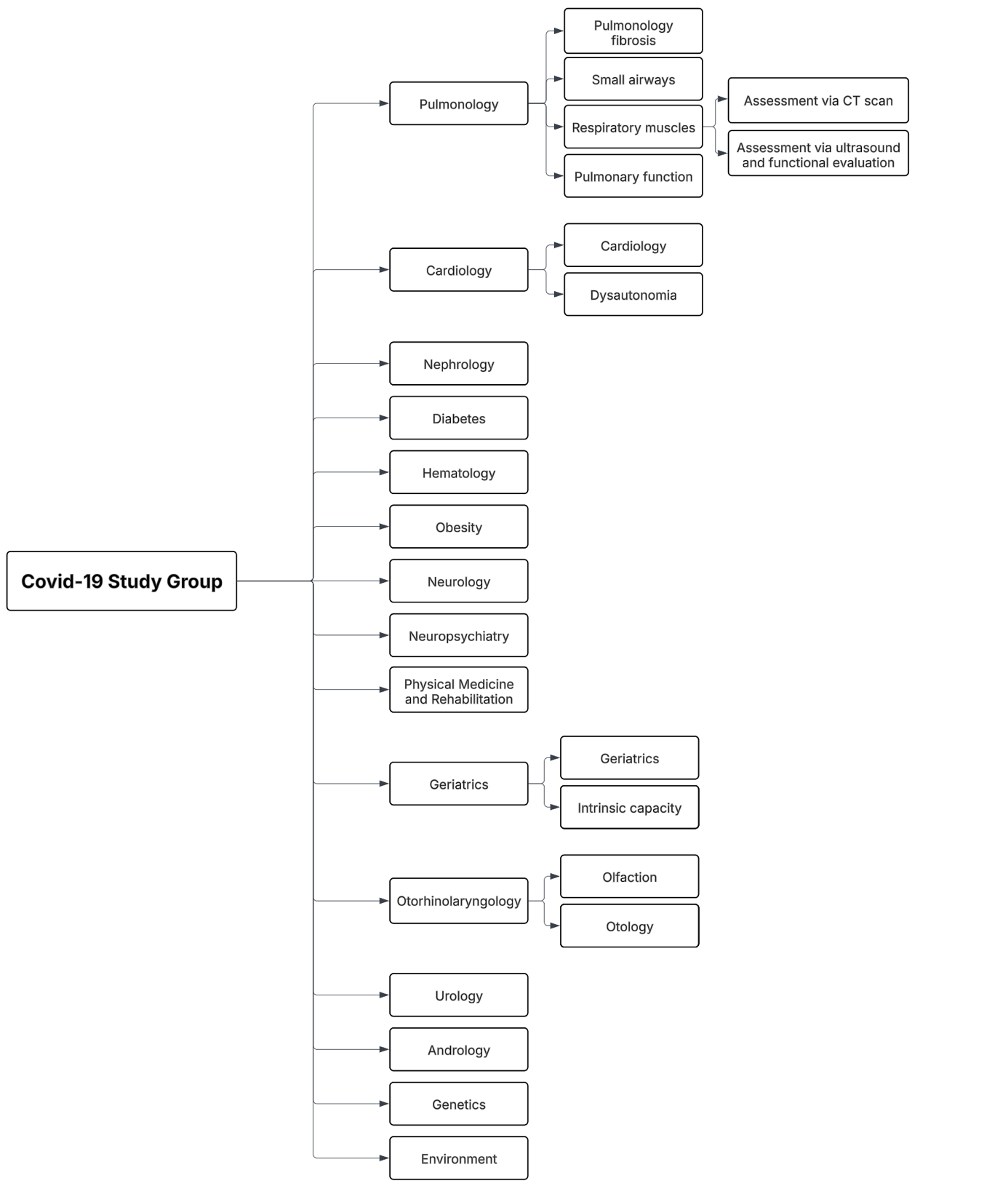
***

**Supplementary figure 1*:*** COVID-19 Study Group diagram.

**Supplementary table 1: Comparison of hospitalization and first follow-up (6–12 months) sociodemographic and clinical variables between participants and non-participants of the third follow-up (41–47 months).**

|  | **Non participants**  **(n=169)** | **Participants**  **(n=523)** | **p-value** |
| --- | --- | --- | --- |
| **Demographics** | | | |
| Age, mean ∓ SD, y | 63 (50, 71) | 60 (49, 69) | 0.4 |
| Male | 87 (51%) | 274 (52%) | 0.8 |
| Race | | | 0.4 |
| White | 23 (14%) | 75 (14%) |  |
| Black | 67 (40%) | 186 (36%) |  |
| Pardo^a^ | 74 (45%) | 238 (46%) |  |
| Asian | 2 (1.2%) | 8 (1.5%) |  |
| Indigenous | 0 (0%) | 6 (1.1%) |  |
| Unknown | 0 (0%) | 9 (1.7) |  |
| Education Level | | | 0.008 |
| Not literate | 10 (6.1%) | 21 (4.0%) |  |
| Elementary Education incomplete | 68 (41%) | 143 (27%) |  |
| Elementary Education complete | 18 (11%) | 61 (12%) |  |
| Secondary Education incomplete | 13 (7.9%) | 36 (6.9%) |  |
| Secondary Education complete | 37 (22%) | 154 (30%) |  |
| Higher Education incomplete | 7 (4.2%) | 30 (5.7%) |  |
| Higher Education complete | 12 (7.3%) | 53 (10%) |  |
| BMI, median (IQR), kg/m² | 31 (28, 35) | 31 (28, 36) | 0.3 |
| **Hospitalization** | | | |
| ICU stay | 106 (63%) | 308 (59%) | 0.4 |
| Hospital LOS, median (IQR), d | 13 (7, 27) | 12 (7, 22) | 0.4 |
| Need for intubation | 71 (42%) | 222 (42%) | >0.9 |
| D Dimer 72h, median (IQR), n-ng/ml | 1,285 (692, 3,308) | 1,115 (651, 3,311) | 0.3 |
| CRP 72h, median (IQR), n-ng/ml | 106 (58, 211) | 126 (66, 214) | 0.2 |
| Severity of acute illness using the WHO clinical progression scale | | | 0.7 |
| Group 1^b^ | 21 (12%) | 53 (10%) |  |
| Group 2^b^ | 69 (41%) | 229 (44%) |  |
| Group 3^b^ | 8 (4.7%) | 19 (3.6%) |  |
| Group 4^b^ | 71 (42%) | 222 (42%) |  |

^a^ Pardo is the exact term used in Brazilian Portuguese, meaning “mixed ethnicity,” according to the

Brazilian Institute of Geography and Statistics. ^b^ Hospitalization Severity Groups: Group 1 – Categories 3 and 4: Patients who did not require any type of oxygen supplementation during hospitalization; Group 2 – Category 5: Patients who required oxygen supplementation only (via nasal cannula or mask) during hospitalization; Group 3 – Category 6: Patients who required non-invasive ventilatory support during hospitalization (high-flow nasal cannula or non-invasive ventilation); Group 4 – Categories 7, 8, and 9: Patients who required any type of invasive ventilatory or organ support, including invasive mechanical ventilation, ECMO, and/or dialysis.SD – standard deviation, IQR – interquartile range, y – years, BMI - body mass index, ICU - Intensive Care Unit, LOS - Length of Stay, CRP - C-reactive protein.

**Supplementary table 2: Comparison of sociodemographic and clinical variables collected during hospitalization and at the first follow-up (6–12 months after discharge) between post-COVID-19 patients who participated in the teleconsultation of the third follow-up (41–47 months) but did not complete all study stages, and those who completed the teleconsultation as well as both in-person visits of the third follow-up.**

|  | **Participated only in the teleconsultation**  **(n=156)** | **Participated in the teleconsultation and in-person visits (n=367)** | **p-value** |
| --- | --- | --- | --- |
| **Demographics** |  |  |  |
| Age, mean ∓ SD, y | 57 (47, 69) | 61 (51, 70) | 0.1 |
| Male | 73 (47%) | 201 (55%) | 0.095 |
| Race | | | 0.9 |
| White | 24 (15%) | 51 (14%) |  |
| Black | 56 (36%) | 130 (36%) |  |
| Pardo^a^ | 72 (46%) | 166 (45%) |  |
| Asian | 2 (1.3%) | 6 (1.6%) |  |
| Indigenous | 1 (0.6%) | 5 (1.4%) |  |
| Education Level | | | 0.10 |
| Not literate | 9 (5.8%) | 12 (3.3%) |  |
| Elementary Education incomplete | 46 (29%) | 97 (27%) |  |
| Elementary Education complete | 22 (14%) | 39 (11%) |  |
| Secondary Education incomplete | 12 (7.7%) | 24 (6.6%) |  |
| Secondary Education complete | 37 (24%) | 117 (32%) |  |
| Higher Education incomplete | 13 (8.3%) | 17 (4.6%) |  |
| Higher Education complete | 17 (11%) | 60 (16%) |  |
| BMI, median (IQR), kg/m² | 31 (28, 38) | 31 (28, 36) | 0.3 |
| **Hospitalization** | | | |
| ICU stay | 85 (54%) | 223 (61%) | 0.2 |
| Hospital LOS, median (IQR), d | 12 (7, 21) | 12 (7, 23) | 0.2 |
| Need for intubation | 60 (38%) | 162 (44%) | 0.2 |
| D Dimer 72h, median (IQR), n-ng/ml | 1,172 (630, 2,515) | 1,109 (660, 3,416) | 0.8 |
| CRP 72h, median (IQR), n-ng/ml | 123 (60, 219) | 128 (69, 212) | 0.4 |
| Severity of acute illness using the WHO clinical progression scale | | | 0.3 |
| Group 1^b^ | 21 (13%) | 32 (8.7%) |  |
| Group 2^b^ | 69 (44%) | 160 (44%) |  |
| Group 3^b^ | 6 (3.8%) | 13 (3.5%) |  |
| Group 4^b^ | 60 (38%) | 162 (44%) |  |

^a^ Pardo is the exact term used in Brazilian Portuguese, meaning “mixed ethnicity,” according to the

Brazilian Institute of Geography and Statistics. ^b^ Hospitalization Severity Groups: Group 1 – Categories 3 and 4: Patients who did not require any type of oxygen supplementation during hospitalization; Group 2 – Category 5: Patients who required oxygen supplementation only (via nasal cannula or mask) during hospitalization; Group 3 – Category 6: Patients who required non-invasive ventilatory support during hospitalization (high-flow nasal cannula or non-invasive ventilation); Group 4 – Categories 7, 8, and 9: Patients who required any type of invasive ventilatory or organ support, including invasive mechanical ventilation, ECMO, and/or dialysis.SD – standard deviation, IQR – interquartile range, y – years, BMI - body mass index, ICU - Intensive Care Unit, LOS - Length of Stay, CRP - C-reactive protein.
